# Predictors of short-term functional recovery in ischemic stroke rehabilitation at community hospitals in Singapore

**DOI:** 10.3389/fstro.2025.1704636

**Published:** 2025-12-16

**Authors:** Wei Na Lai, Cheryl Yan Fang Tan, Chong Yau Ong, Michelle Shu Jing Wong, Lian Leng Low

**Affiliations:** 1 Post - Acute and Continuing Care, Singhealth Community Hospitals, Singapore, Singapore; 2 Research & Translational Innovation Office, Singhealth Community Hospitals, Singapore, Singapore; 3 Singhealth Community Hospitals, Singapore, Singapore; 4 SingHealth Regional Health System, Singapore, Singapore

**Keywords:** community hospitals, early rehabilitation, functional recovery, predictors of outcome after stroke, stroke rehabilitation and recovery

## Abstract

**Objectives:**

Stroke remains a leading cause of death and disability worldwide. While functional outcome predictors are well established in acute rehabilitation settings, less is known in community hospitals, which typically manage stroke patients with moderate or isolated impairments. This study aimed to identify predictors of short-term functional improvement in stroke survivors admitted to community hospitals in Singapore.

**Design:**

Prospective cohort study.

**Setting and participants:**

The study included 216 stroke survivors admitted to Outram and Sengkang Community Hospitals for inpatient rehabilitation.

**Methods:**

Functional status was measured using the Modified Barthel Index (MBI) on admission and discharge. Data on depressive symptoms (PHQ-2), resilience (CD-RISC-10), comorbidities, stroke severity (NIHSS), time to rehabilitation initiation, and sociodemographics were collected. Logistic regression identified predictors of significant functional improvement, defined as at least a one-level increase in MBI.

**Results:**

Participants' mean age was 71.20 years; most were male (59.30%), Chinese (82.00%), unemployed (58.80%), and living with family (86.50%). Functional improvement was more likely among those who were premorbidly independent (65.70%), had mild depressive symptoms (PHQ-2 ≤ 2; 63.70%), experienced mild strokes (NIHSS ≤ 4; 43.10%), or started rehabilitation within 1 day of onset (33.80%). Older age (*p* = 0.02) and shorter time to rehabilitation (*p* = 0.03) independently predicted functional improvement.

**Conclusion and implications:**

Older age and early rehabilitation were significantly associated with greater short-term functional gains in community hospital stroke survivors, underscoring the importance of timely rehabilitation to optimize recovery after stroke, even for older adults.

## Introduction

Stroke remains a major global health concern, ranking as the second leading cause of death and third in terms of disease burden (GBD 2019 Stroke Collaborators, 2021). Annually, around 15 million people experience a stroke, with 5 million resulting in death and another 5 million living with permanent disabilities. This imposes a substantial emotional and financial burden on individuals, families, and healthcare systems (World Health Organization, 2022a). In Singapore, stroke is the fourth leading cause of death, responsible for 6% of all mortalities. National stroke episodes have increased from 5,890 in 2010 to 8,849 in 2019, with a mean age of onset at 65 years (National Registry of Diseases Office, 2022). As the population ages, the national stroke burden is expected to rise (Chen, 2008). Over half of stroke survivors experience ongoing motor impairments, affecting their functional independence and quality of life (Mayo et al., 1999). Understanding factors associated with recovery is vital for optimizing rehabilitation and post-acute care planning.

Understanding which factors influence recovery is therefore crucial for tailoring rehabilitation programs and improving patient outcomes. Over the past decades, numerous studies in acute inpatient settings have identified clinical and demographic predictors of functional outcomes, stroke severity, and previous stroke history (Meyer et al., 2015). However, these findings are largely derived from acute hospital populations, and much less is known about such predictors in community or step-down rehabilitation settings, where patients often have stabilized medical conditions but ongoing functional limitations. In one local study conducted in a dedicated rehabilitation unit within a tertiary academic acute hospital, stroke type (especially haemorrhagic and anterior circulation infarcts) predicted longer acute length of stay, while functional score on admission, urinary tract infection, depression, lack of caregiver, and male gender predicted longer rehabilitation stay, with poor correlation between acute and rehab durations (Ng et al., 2016).

**Figure 1:**
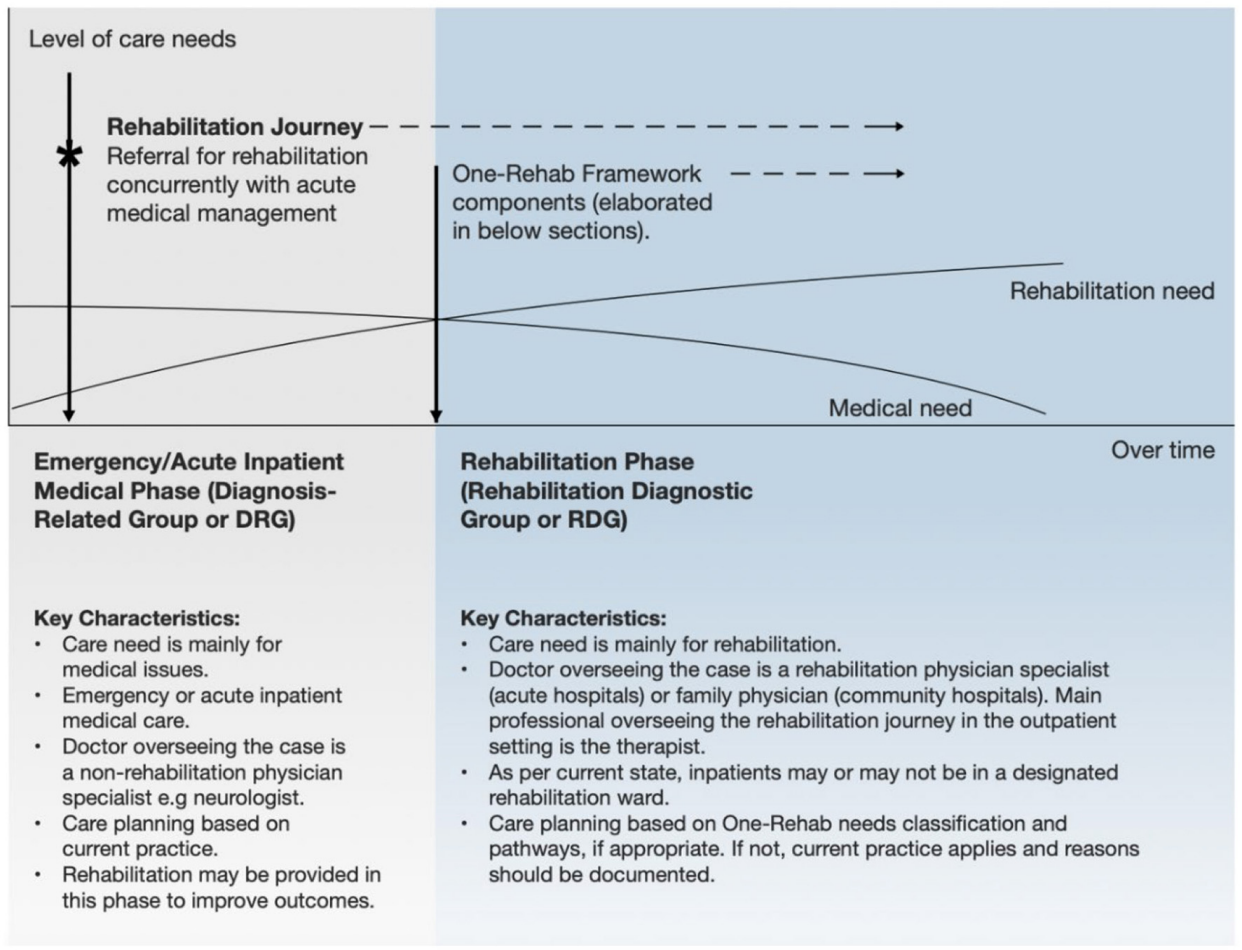
The rehabilitation phase in community hospitals follows the emergency and acute inpatient phase. Source: Ministry of Health Singapore (2022).

**Figure 2:**
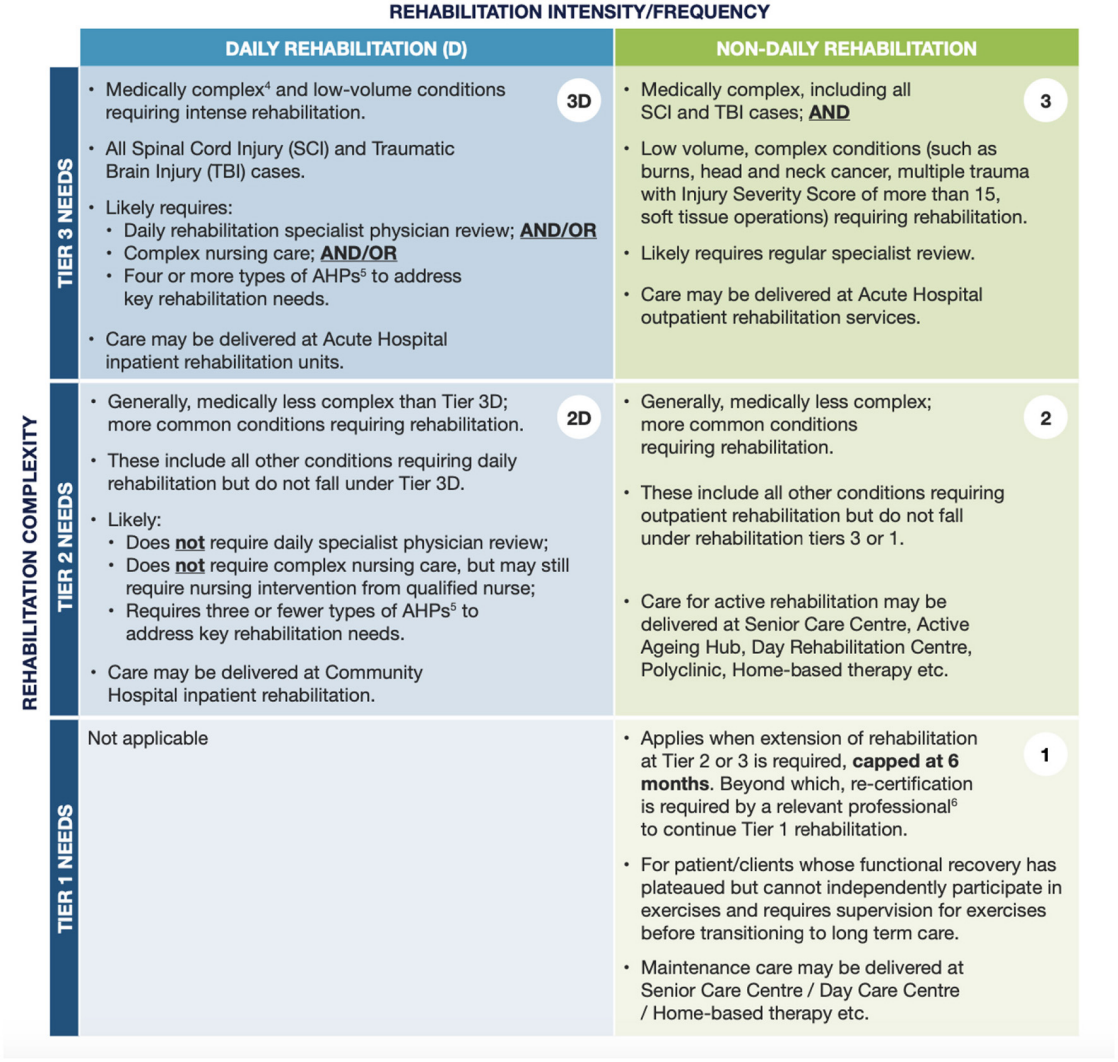
Rehabilitation complexity tier and rehabilitation intensity. ^4^The definitions of medically complex conditions and complex nursing care will be deferred to the healthcare professionals within the cluster and organization. ^5^Includes physiotherapist, occupational therapist, speech therapist, dietitian, clinical psychologist, podiatrist etc. Number of therapists should not singly distinguish rehabilitation care needs of different tiers. ^6^Therapist with full AHPC registration; OR Advanced Practice Nurse registered with Singapore Nursing Board; OR Medical practitioner with conditional or full registration under Singapore Medical Council. Source: Ministry of Health Singapore (2022).

Our study focuses on community hospitals in Singapore—step-down facilities providing multidisciplinary rehabilitation once acute medical care has stabilized, in accordance with the National One-Rehabilitation Framework (One-Rehab) guidelines (Figure 1). These institutions typically manage stroke patients with moderate impairments or isolated deficits who benefit from daily therapy but do not require intensive inpatient rehabilitation (Figure 2; Ministry of Health Singapore, 2022).

Functional improvement during this subacute phase is important for discharge planning, caregiver preparation, and determining the need for ongoing rehabilitation. Furthermore, early gains are also linked to better long-term recovery (Wang et al., 2011; Mutai et al., 2012). Yet, few studies in Singapore have examined short-term functional improvement in community hospitals, despite their critical role in stroke care.

To address this gap, we adopted the Social Determinants of Health (SDoH) framework to investigate how medical, sociodemographic, and psychological factors interact to influence recovery (World Health Organization, 2022b). SDoH encompasses a range of individual and contextual influences, including income, education, social networks, and healthcare access, which may impact rehabilitation outcomes.

The primary aim of this study was to identify predictors of short-term functional improvement among stroke survivors admitted to community hospitals. Functional improvement was defined as a one-level increase on the Modified Barthel Index (MBI), which indicates a clinically meaningful shift in independence across activities of daily living, with potential implications for independence and self-care. Our institution employs the Modified Barthel Index (MIBI) as its standard measure of functional independence in community hospitals (Htun et al., 2022). The MIBI has been validated in diverse rehabilitation populations and shows strong correlations with the FIM, capturing similar domains of self-care and mobility. Because it is integrated into our clinical workflow and routinely documented, use of the MIBI ensured complete and consistent data across all participants (Campagnini et al., 2022; Htun et al., 2022; Shah et al., 1989).

## Methodology

### Study design and site

This prospective cohort study was conducted from March 2022 to April 2024 at Outram Community Hospital (OCH) and Sengkang Community Hospital (SKCH), both operated by SingHealth Community Hospitals (SCH; SingHealth Community Hospitals, 2022). OCH is a 382-bed facility co-located with Singapore General Hospital (SGH), which manages a large portion of stroke patients nationally (Singapore General Hospital, 2022). SKCH, with 247 beds, is situated next to Sengkang General Hospital (SKGH) and primarily receives referrals from SKGH (SingHealth Community Hospitals, 2022). Both hospitals provide an average inpatient rehabilitation duration of 26 days, supported by a multidisciplinary team including physicians, nurses, physiotherapists, occupational therapists, speech therapists, dietitians, and pharmacists.

### Participants

Eligible participants included medically stable ischemic stroke patients capable of participating in rehabilitation and providing informed consent.

Exclusion criteria were:

- Haemorrhagic stroke,
- Cognitive impairment (Abbreviated Mental Test score < 7),
- Inability to communicate by any means,
- Refusal or inability to consent.

All questionnaires were administered by trained interviewers.

### Variables and data collection

Guided by the SDoH framework, we categorized potential predictors into three domains:

1) Medical factors: comorbidities (such as hypertension, hyperlipidaemia, diabetes mellitus, ischemic heart disease), prior stroke history, stroke severity (NIHSS), time from stroke onset to rehabilitation initiation, and length of hospital stay.
2) Socio-demographic factors: age, sex, ethnicity, marital status, level of education and employment status, smoking and alcohol consumption, living arrangement, caregiver presence, premorbid functional status.
3) Psychosocial factors: depression and resilience.

Cognitive function was assessed using the Abbreviated Mental Test (AMT), a 10-item tool measuring orientation, memory, attention, and general knowledge (Hodkinson, 2012). A score ≤ 6 suggests cognitive impairment, with high sensitivity of 70%−80% and specificity of 71%−90% (MacKenzie et al., 1996).

Depression was screened using the Patient Health Questionnaire-2 (PHQ-2**)**, which evaluates mood and anhedonia over the past 2 weeks. A score ≥3 was used as the cutoff, consistent with validated thresholds for detecting depressive disorders (Kroenke et al., 2003).

Resilience was measured using the Connor-Davidson Resilience Scale (CD-RISC-10), a 10-item scale assessing adaptability, problem-solving ability, emotional regulation, response to failure, and stress coping. Scores range from 0 to 40, with higher scores indicating greater resilience. The CD-RISC-10 has demonstrated strong psychometric properties, including internal consistency, test–retest reliability, and both convergent and divergent validity (Campbell-Sills et al., 2009; Riopel, 2010). It was used with permission from the original authors.

Functional outcomes were assessed using the Modified Barthel Index (MBI), which evaluates independence in 10 activities of daily living (ADLs; Shah et al., 1989; Mahoney and Barthel, 1965).

The total summation score ranges from 0 to 100, which corresponds to the following dependency levels:

0–20 Total dependence
21–60 Severe dependence
61–90 Moderate dependence
91–99 Slight dependence
100 Independence

The MBI demonstrates strong reliability and validity, with high internal consistency (α = 0.90), test-retest reliability (*r* = 0.89), and good convergent validity (*r* = 0.74–0.80) with other physical disability measures (O'Sullivan and Schmitz, 2022). The MBI is the standard measure used in Singapore community hospitals for rehabilitation assessment (Chen et al., 2013).

### Statistical analysis

#### Sample size

We calculated the sample size based on the rule of 10 events per variable in regression analysis. With 18 variables, we required 180 participants, increasing this to 216 to account for a 20% potential dropout (Peduzzi et al., 1996; Concato et al., 1995).

### Analytical methods

Descriptive statistics summarized baseline characteristics. Functional improvement was defined as a one-level increase in MBI dependency level from admission to discharge or Day 26. Participants were divided into two groups based on improvement status:

1. Those with significant improvement in MBI
2. Those without significant improvement

Continuous variables were analyzed using binary logistic regression, and categorical variables were analyzed using Chi-square or Fisher's exact tests. Multivariate logistic regression identified independent predictors of improvement, with *p*-values < 0.05 considered significant.

Data analysis was performed using SPSS version 26.0 (IBM Corp., Armonk, NY, United States).

### Ethics approval

This study received ethics approval from the SingHealth Centralized Institutional Review Board (CIRB Ref: 2022/2110).

## Results

Baseline demographic and clinical characteristics of participants are summarized in Table 1. The mean age was 71.18 years (SD 10.70). Most were male (59.30%), Chinese (82.00%), married (59.70%), and living with family (86.50%). A majority were retired or unemployed (58.80%). Most had comorbidities (90.70%) but no prior stroke (79.10%) and were non-smokers (76.40%) and non-drinkers (80.10%).

**Table 1:** Demographic, clinical characteristics, and functional status of participants.

**Variables**	**Participants with significant improvement in MBI *n* = 150 (%)**	**Participants without significant improvement in MBI *n* = 66 (%)**	***p*-value**
Age (years ± SD)	70.05 ± 10.81	73.73 ± 10.12	**0.02**
**Age**			
< 65 years	108 (72.00)	56 (84.85)	**0.04**
≥65 years	42 (28.00)	10 (15.15)	
**Gender**			
Male	87 (58.00)	41 (62.12)	0.57
Female	63 (42.00)	25 (37.88)	
**Race**			
Chinese	122 (81.33)	55 (83.33)	0.20
Malay	20 (13.33)	8 (12.12)	
Indian	6 (4.00)	2 (3.03)	
Others	2 (1.33)	1 (1.52)	
**Marital status**			
Single	16 (10.67)	13 (19.70)	0.26
Married	92 (61.33)	37 (56.06)	
Divorced	13 (8.67)	3 (4.54)	
Widowed	29 (19.33)	13 (19.70)	
**Living arrangement**			
Alone	16 (10.67)	13 (19.70)	0.07
Family/friends	134 (89.33)	53 (80.30)	
**Presence of caregiver**			
Yes	95 (63.33)	43 (65.15)	0.40
No	55 (36.67)	23 (34.85)	
**Education level**			
No formal education	15 (10.00)	11 (16.67)	0.18
Primary	55 (36.67)	21 (31.82)	
Secondary	39 (26.00)	15 (22.73)	
Tertiary	41 (27.33)	19 (28.79)	
**Occupation**			
Working full-time/part-time	53 (35.33)	16 (24.24)	0.82
Retired and/or unemployed	85 (56.67)	42 (63.64)	
Homemaker	12 (8.00)	8 (12.12)	
**Premorbid functional status**			
Independent	142 (94.66)	58 (87.88)	0.11
Partially dependent	7 (4.67)	8 (12.12)	
Fully dependent	1 (0.67)	0 (0.00)	
**PHQ-2 total score**			
2 or less	136 (90.67)	55 (83.33)	0.12
3 or greater	14 (9.33)	11 (16.67)	
**Previous stroke**			
Yes	29 (19.33)	16 (24.24)	0.41
No	121 (80.67)	50 (75.76)	
**Presence of** ^*^comorbidities			
Present	134 (89.33)	62 (93.94)	0.28
Absent	16 (10.67)	4 (6.06)	
**Number of comorbidities**			
< 5	101 (67.33)	45 (68.18)	0.73
5–10	49 (32.67)	21 (31.82)	
**NIHSS severity (categories)**			
Mild (1–4)	93 (62.00)	38 (57.58)	0.53
Mild to moderately severe (5–14)	50 (33.33)	27 (40.90)	
Severe (15–24)	6 (4.00)	1 (1.52)	
Very severe (>25)	1 (0.67)	0 (0.00)	
**Time between onset of stroke and initiation of rehabilitation (day)**			
0	7 (4.67)	5 (7.58)	0.20
1	73 (48.67)	38 (57.57)	
2	38 (25.33)	17 (25.76)	
3	27 (18.00)	6 (9.09)	
4	5 (3.33)	0 (0.00)	
**MBI on admission**			
Slight dependence	0 (0.00)	1 (1.52)	0.10
Moderate dependence	40 (26.67)	12 (18.18)	
Severe dependence	97 (64.66)	42 (63.64)	
Total dependence	13 (8.67)	11 (16.66)	
**Smoking**			
Yes	20 (13.33)	9 (13.64)	0.53
No	117 (78.00)	48 (72.72)	
Ex-smoker	13 (8.67)	9 (13.64)	
**Alcohol consumption**			
Yes	19 (12.67)	12 (18.18)	0.54
No	122 (81.33)	51 (77.27)	
Ex-drinker	9 (6.00)	3 (4.55)	
CD-RISC-10 score (mean ± SD)	25.81 ± 6.71	25.38 ± 7.41	0.67
Length of stay (mean ± SD)	28.49 ± 16.46	32.29 ± 21.34	0.16

CD-RISC-10, Conner-Davidson Resilience Score 10-item; MBI, modified Barthel Index; NIHSS, National Institutes of Health Stroke Scale; PHQ-2, Patient Health Questionnaire-2.

^*^Comorbidities: hypertension, diabetes mellitus, ischemic heart disease, atrial fibrillation (Simić-Panić, 2018; Karatepe et al., 2008).

The bold values indicate significance with *p* < 0.05.

Participants who demonstrated significant functional improvement were more likely to be premorbidly independent (94.70% vs. 87.88%, *p* = 0.11), have a PHQ-2 score ≤ 2 (90.67% vs. 87.33%, *p* = 0.12), and a mild stroke severity by NIHSS (62.00% vs. 57.58%, *p* = 0.53); however, these were not statistically significant.

The average CD-RISC-10 score was 25.68 (SD 6.92). Participants who improved had slightly higher scores (25.81 ± 6.71) than those who did not (25.38 ± 7.41), but this difference was not significant (*p* = 0.67).

The mean length of stay was 29.65 days (SD 18.1). Participants who improved functionally had shorter hospital stays (28.49 ± 16.46 vs. 32.29 ± 21.34 days, *p* = 0.16), though this was also not significant.

Univariate analysis (Table 2) revealed two significant predictors for functional improvement at Day 26 post-rehabilitation:

- Age 65 years and above (OR = 0.46, 95% CI: 0.21–1.00, *p* = 0.02).
- Rehabilitation within 2 days of stroke onset (OR = 0.37, 95% CI: 0.15–0.93, *p* = 0.04).

**Table 2:** Univariate analysis of factors associated with significant improvement in MBI.

**Variables**	**Unadjusted odds-ratio (95% CI)**	***p*-value**
**Age (years)**		
< 65	0.46 (0.21–1.00)	**0.04**
≥65	Ref	
**Gender**		
Female	0.84 (0.47–1.53)	0.57
Male	Ref	
**Race**		
Non-Chinese	0.87 (0.41–1.88)	0.73
Chinese	Ref	
**Marital status**		
Single	3.20 (0.89–4.61)	0.10
Widowed	1.12 (0.52–2.38)	0.78
Divorced	0.57 (0.16–2.13)	0.41
Married	Ref	
**Occupation**		
Working full-time/part-time	0.61 (0.31–1.19)	0.15
Homemaker	1.35 (0.51–3.55)	0.54
Retired and/or unemployed	Ref	
**Living arrangement**		
Alone	2.05 (0.93–4.56)	0.08
Family/friends	Ref	
**Education level**		
No formal education	1.92 (0.76–4.85)	0.17
Secondary	1.01 (0.46–2.20)	0.98
Tertiary	1.21 (0.58–2.55)	0.61
Primary	Ref	
**Premorbid functional status**		
Partially/fully dependent	2.45 (0.88–6.83)	0.09
Independent	Ref	
**PHQ-2 total score**		
3 or greater	1.94 (0.83–4.54)	0.13
2 or less	Ref	
**Previous stroke**		
Yes	1.34 (0.67–2.67)	0.41
No	Ref	
**Presence of** ^*^comorbidities		
Present	1.85 (0.59–5.77)	0.29
Absent	Ref	
**NIHSS severity (categories)**		
Mild to moderately severe (5–14)	1.32 (0.72–2.41)	0.36
Severe (15–24)/very severe (>25)	0.35 (0.04–2.94)	0.33
Mild (1–4)	Ref	
**Time between onset of stroke and initiation of rehabilitation**		
**(day)**		
3 or more	0.37 (0.15–0.93)	**0.04**
2 or less	Ref	
**MBI on admission**		
Slight/moderate dependence	0.75 (0.36–1.55)	0.44
Total dependence	1.95 (0.81–4.72)	0.14
Severe dependence	Ref	
**Smoking**		
Yes	1.10 (0.47–2.58)	0.83
Ex-smoker	1.69 (0.68–4.21)	0.26
No	Ref	
**Alcohol consumption**		
Yes	1.51 (0.68–3.34)	0.31
Ex-drinker	0.80 (0.21–3.07)	0.74
No	Ref	
**CD-RISC-10 score**	0.99 (0.95–1.03)	0.67
**Length of stay**	1.01 (0.10–1.03)	0.16

CD-RISC-10, Conner-Davidson Resilience Score 10-item; MBI, modified Barthel Index; NIHSS, National Institutes of Health Stroke Scale; PHQ-2, Patient Health Questionnaire-2.

^*^Comorbidities: hypertension, diabetes mellitus, ischemic heart disease, atrial fibrillation.

The bold values indicate significance with *p* < 0.05.

Multivariate analysis (Table 3) confirmed similar associations after adjusting for covariates, including depression (PHQ-2), history of previous stroke, initial stroke severity (NIHSS), and functional status upon admission (MBI).

- Younger age (less than 65 years) was associated with lower odds of significant functional improvement (adjusted OR = 0.42, 95% CI: 0.19–1.00, *p* = 0.03).
- Late rehabilitation (on or after Day 3 of stroke onset) was associated with lower odds of improvement (adjusted OR = 0.36, 95% CI: 0.14–1.00, *p* = 0.04).

**Table 3:** Multivariate analysis of factors associated with significant improvement in MBI.

**Variables**	**Adjusted odds-ratio (95% CI)**	***p*-value**
**Age (years)**		
< 65	0.42 (0.19–1.00)	**0.03**
≥65	Ref	
**PHQ-2 total score**		
3 or greater	1.96 (0.80–4.83)	0.14
2 or less	Ref	
**Previous stroke**		
Yes	0.96 (0.43–2.14)	0.93
No	Ref	
**NIHSS severity (categories)**		
Mild to moderately severe (5–14)	1.21 (0.62–2.36)	0.58
Severe (15–24)/very severe (>25)	0.46 (0.05–4.00)	0.48
Mild (1–4)	Ref	
**Time between onset of stroke and rehab admission (day)**		
3 or more	0.36 (0.14–1.00)	**0.04**
2 or less	Ref	
**MBI on admission**		
Slight/moderate dependence	0.80 (0.38–1.70)	0.56
Total dependence	2.01 (0.76–5.36)	0.16
Severe dependence	Ref	

MBI, modified Barthel Index; NIHSS, National Institutes of Health Stroke Scale; PHQ-2, Patient Health Questionnaire-2.

The bold values indicate significance with *p* < 0.05.

Admission MBI scores also showed a trend—those with total dependence had higher odds of improvement, but this was not statistically significant (adjusted OR = 2.01, 95% CI: 0.76–5.36, *p* = 0.16).

## Discussion

Our study identified two significant predictors of short-term functional improvement in stroke survivors admitted to community hospitals in Singapore: older age (defined as ≥65 years) and early initiation of rehabilitation (within 2 days of stroke onset). “Short-term functional improvement” refers to within a typical single inpatient rehabilitation episode which is about 4 weeks. This is consistent with other studies in subacute or community rehabilitation that focus on outcomes measured at discharge rather than months later (Htun et al., 2022). These findings are especially relevant given the unique population and care model of community hospitals, which cater to patients with moderate deficits or isolated impairments who do not require intensive acute rehabilitation. Compared to acute hospital settings, community hospitals provide a more resource-conscious but still structured environment for recovery and understanding predictors of improvement within this context is essential for optimizing care delivery.

Older age as a predictor of functional improvement

Contrary to the common belief that older age is associated with poorer rehabilitation outcomes, our findings showed that older adults had better short-term functional improvement. This may be explained by local referral patterns—younger stroke survivors referred to community hospitals often have more severe strokes with poorer prognoses, which may limit their rehabilitation potential. This underscores the rationale for frameworks like the One-Rehab guidelines, which aim to enhance rehabilitation efficiency in Singapore's healthcare system (Ministry of Health Singapore, 2022).

Younger patients in community hospitals may represent a subgroup with more complex stroke etiologies, such as arterial dissections, congenital heart conditions, autoimmune disorders, or clotting abnormalities. These conditions can contribute to greater stroke severity and poorer recovery. In addition, lifestyle-related risk factors (e.g., smoking, obesity, hyperlipidaemia) may exacerbate stroke severity in younger individuals, further complicating recovery (UCHealth, 2019; Blocker, 2023). Supporting this, prior studies have found that while older patients (≥60 years) with moderate to moderately severe strokes show substantial improvements during inpatient rehabilitation, younger age, greater stroke severity, and lower admission function were associated with longer hospital stays (Albu et al., 2024). Other studies also note that while older patients may have worse discharge outcomes, age has less impact when functional gains are measured after a defined rehabilitation period (Lehmann et al., 1975). Kalra (1994) further demonstrated that intensive stroke rehabilitation benefits all age groups without requiring additional resources.

However, age-related gains may not persist over time. One study found that stroke patients over 70 showed significant subacute recovery, but many experienced functional decline between 18 and 60 months post-stroke (Shin et al., 2022). This emphasizes the need for age-tailored strategies: early intensive rehabilitation for younger patients, and ongoing monitoring and support for older patients to sustain functional gains (Yoo et al., 2020).

Early rehabilitation

Our study also supports the growing body of evidence emphasizing the importance of early rehabilitation. Initiating rehabilitation within 2 days of stroke onset was associated with better functional outcomes—echoing findings by Otokita et al. (2021), who found early rehabilitation improved discharge outcomes in ischemic stroke patients. This approach is also endorsed by international guidelines, such as the European Stroke Organization and the American Heart Association/American Stroke Association, which recommend early discharge to community-based rehabilitation for medically stable patients with mild-to-moderate strokes (Powers et al., 2019; Cerebrovascular Diseases Working Group, 2008). Early rehabilitation may enhance neuroplasticity, restore brain function, and prevent complications associated with immobility. In Singapore, these findings support streamlined workflows to facilitate early transfer (within 48 h) from acute hospitals to community care, along with standardized rehabilitation pathways to minimize care variation and optimize recovery.

Depression

We did not find a significant association between PHQ-2 scores and short-term functional improvement, in contrast with previous studies reporting poorer recovery among stroke patients with depression (Van De Weg et al., 1999; Sloane et al., 2023). Most of our participants had PHQ-2 scores ≤ 2, suggesting minimal depressive symptoms. Some literature indicates that only major depression, rather than subclinical symptoms, predicts poorer functional outcomes (Pohjasvaara et al., 2001). This discrepancy may reflect Singapore's unique healthcare context, including integrated care pathways, strong social support systems, and cultural attitudes toward mental health. Additionally, PHQ-2 may lack the sensitivity of PHQ-9 in detecting major depressive disorder (Arroll et al., 2010). Furthermore, the multidisciplinary care model used in community hospitals may help buffer the impact of depression on functional recovery (Hadidi et al., 2009).

Psychological resilience

Consistent with Gyawali et al. (2020), our study found no significant association between psychological resilience (CD-RISC-10) and short-term functional improvement. However, resilience may play a greater role in cognitive outcomes post-stroke, suggesting a need for further research in this area (Gyawali et al., 2020).

The mean CD-RISC-10 score in our sample was 25.68 ± 6.92, consistent with findings from Campbell-Sills and Stein (2007) and Serrano-Parra et al. (2013), as well as a Singapore study reporting an average score of 26.50 among older adults (Bautista et al., 2018) Notably, lower resilience scores were seen among individuals with physical illnesses, including stroke. While our participants generally displayed moderate resilience, resilience-building interventions may still help patients cope more effectively with post-stroke challenges (Southwick et al., 2014).

Other predictors

Unlike earlier studies, we found no significant association between functional improvement and previous stroke history, initial stroke severity, or admission functional scores. This differs from research by Wang et al. (2011), Duncan et al. (2000), and Bang et al. (2005), who identified these factors as key predictors of long-term recovery (Bautista et al., 2018; Southwick et al., 2014). Several factors may explain this divergence. First, neuroplasticity allows functional recovery even in patients with severe strokes (Inouye et al., 2000; FlintRehab, 2023). Research by Dobkin (2005) and Teasell et al. (2005) supports the idea that intensive, individualized rehabilitation can yield meaningful improvement regardless of initial deficits. Recovery is likely influenced by a complex interplay of factors—such as depression, cognitive status, age, and timeliness of intervention—rather than any single predictor alone.

## Strengths and limitations

Our study offers several strengths. First, the community hospital (transitional care) setting provides unique insight into the recovery of stroke survivors with moderate deficits (Tier 2D). The inclusion of psychosocial variables, such as depression and resilience, reflects a comprehensive approach to evaluating stroke recovery. The multidisciplinary team-based approach also ensures holistic rehabilitation addressing physical, cognitive, and emotional needs, while strong community linkages support successful transitions to home. Second, the study had a low dropout rate of just 2% (4 out of 216 participants), enhancing statistical power and reducing attrition bias. Most participants completed their rehabilitation and were successfully discharged home. Third, by identifying factors associated with short-term functional improvement, this study provides a foundation for future longitudinal research aimed at establishing causal relationships and exploring underlying mechanisms.

However, several limitations warrant consideration. First, by excluding patients with cognitive impairment, we may have limited the applicability of our findings to the broader stroke population, as cognitive deficits are common post-stroke. Second, our study only examined short-term outcomes; further research is needed to explore the sustainability of early functional gains. Third, we were unable to assess other potentially important variables such as socioeconomic status, intensity and duration of rehabilitation, and family support, which may influence recovery trajectories.

The community hospital model in Singapore operates within a tightly integrated national health system, characterized by high continuity of care, publicly funded rehabilitation subsidies, and short transfer times between acute and post-acute settings under the One-Rehabilitation Framework. Such system-level coordination may not be present in other countries where subacute rehabilitation is delivered through more fragmented, insurance-based or outpatient-dominated pathways (Ifejika et al., 2025). Consequently, the observed benefits of early transfer and structured multidisciplinary rehabilitation may be attenuated in health systems with delayed transitions or variable access to therapy services. Cultural and social factors unique to Singapore such as high family co-residence rates (86.50% in our sample), community-based caregiving expectations, and multi-ethnic but urban population homogeneity, may also influence recovery trajectories differently from Western or rural Asian contexts (Gubhaju et al., 2018).

## Conclusion and future implications

This study provides valuable insights into stroke rehabilitation outcomes within Singapore's community hospital setting. We identified two key predictors of short-term functional improvement: older age and early initiation of rehabilitation. Notably, older patients achieved better functional outcomes than expected, challenging common assumptions about age-related limitations in recovery—an important finding given Singapore's rapidly aging population. Early rehabilitation also proved to be a critical factor, with prompt initiation within 2 days of stroke onset associated with significantly better outcomes. These findings highlight the importance of streamlined care pathways, including timely transfers from acute care to rehabilitation, and support the development of age-specific strategies that prioritize early intervention.

Moving forward, a more comprehensive approach to stroke rehabilitation is needed—one that considers not only traditional clinical indicators but also psychosocial and contextual factors. Future research should extend follow-up beyond the short term and include a wider spectrum of stroke survivors to better inform practices and policies in community-based stroke rehabilitation.

Future studies should aim to validate these findings across multiple community and regional rehabilitation settings to enhance generalisability. Comparative research between Singapore and other health systems could clarify how differences in healthcare financing, rehabilitation intensity, and care coordination influence recovery trajectories. Inclusion of cognitive and socioeconomic variables will allow for a more comprehensive understanding of stroke recovery determinants. Longitudinal studies extending follow-up beyond discharge are needed to assess the sustainability of short-term gains and their translation into long-term independence and community reintegration. In addition, implementation-focused research using frameworks such as RE-AIM or CFIR could examine how organizational processes, referral timing, and resource allocation affect the timeliness and effectiveness of rehabilitation. These efforts will strengthen evidence for optimizing early rehabilitation and support the design of scalable, system-level strategies to improve functional recovery after stroke.

## Data Availability

The raw data supporting the conclusions of this article will be made available by the authors, without undue reservation.

## References

[B1] Albu S. Izcara López De Murillas E. Secanell Espluga M. Jimenez Crespo A. Kumru H. (2024). Clinical profiles and functional outcomes in elderly stroke survivors undergoing neurorehabilitation: a retrospective cohort study. Egypt J. Neurol. Psychiatr. Neurosurg. 60:102. doi: 10.1186/s41983-024-00877-x

[B2] Arroll B. Goodyear-Smith F. Crengle S. Gunn J. Kerse N. Fishman T. . (2010). Validation of PHQ-2 and PHQ-9 to screen for major depression in primary care. Ann. Fam. Med. 8, 348–353. doi: 10.1370/afm.1139 20644190 PMC2906530

[B3] Bang O. Y. Park H. Y. Yoon J. H. Yeo S. H. Kim J. W. Lee M. A. . (2005). Predicting long-term outcome after subacute stroke within the MCA territory. J. Clin. Neurol. 1:148. doi: 10.3988/jcn.2005.1.2.148 20396462 PMC2854920

[B4] Bautista M. A. Li Y. T. Malhotra R. (2018). Caregivers of Older Adults in Singapore: An Empirical Synthesis. Centre for Ageing Research & Education, Duke-NUS Medical School Policy Brief. Available online at: https://www.duke-nus.edu.sg/.../care-research-brief-6-caregivers-of-older-adults-in-singapore.pdf (Accessed August 27, 2024).

[B5] Blocker K. (2023). Strokes in Young People: They're Occurring More Often. Why? Aurora, CO: UCHealth. Available online at: https://www.uchealth.org/today/why-are-strokes-in-young-people-occurring-more-often/ (Accessed July 15, 2024).

[B6] Campagnini S. Liuzzi P. Mannini A. Basagni B. Macchi C. Carrozza M. C. . (2022). Cross-validation of predictive models for functional recovery after post-stroke rehabilitation. J. NeuroEng. Rehabil. 19:96. doi: 10.1186/s12984-022-01075-7 36071452 PMC9454118

[B7] Campbell-Sills L. Forde D. R. Stein M. B. (2009). Demographic and childhood environmental predictors of resilience in a community sample. J. Psychiatr. Res. 43, 1007–1012. doi: 10.1016/j.jpsychires.2009.01.013 19264325

[B8] Campbell-Sills L. Stein M. B. (2007). Psychometric analysis and refinement of the CD-RISC: validation of a 10-item measure of resilience. J. Trauma Stress. 20, 1019–1028. doi: 10.1002/jts.20271 18157881

[B9] Cerebrovascular Diseases Working Group (2008). Guidelines for management of ischaemic stroke and TIA 2008. Cerebrovasc. Dis. 25, 457–507. doi: 10.1159/000131083 18477843

[B10] Chen C. Koh G. C. H. Naidoo N. Cheong A. Fong N. P. Tan Y. V. . (2013). Trends in length of stay, functional outcomes, and discharge destination stratified by disease type for inpatient rehabilitation in Singapore community hospitals from 1996 to 2005. Arch. Phys. Med. Rehabil. 94, 1342–1351.e4. doi: 10.1016/j.apmr.2013.01.006 23333659

[B11] Chen N. V. (2008). Burden of stroke in Singapore. Int. J. Stroke 3, 51–54. doi: 10.1111/j.1747-4949.2008.00181.x 18705915

[B12] Concato J. Peduzzi P. Holford T. R. Feinstein A. R. (1995). Importance of events per independent variable in proportional hazards analysis I. J. Clin. Epidemiol. 48, 1495–1501. doi: 10.1016/0895-4356(95)00510-2 8543963

[B13] Dobkin B. H. (2005). Rehabilitation after stroke. N. Engl. J. Med. 352, 1677–1684. doi: 10.1056/NEJMcp043511 15843670 PMC4106469

[B14] Duncan P. W. Lai S. M. Keighley J. (2000). Defining post-stroke recovery: implications for design and interpretation of drug trials. Neuropharmacology 39, 835–841. doi: 10.1016/S0028-3908(00)00003-4 10699448

[B15] FlintRehab (2023). Neuroplasticity after Stroke: How the Brain Rewires Itself to Recover from Injury. Available online at: https://www.flintrehab.com/neuroplasticity-after-stroke/ (Accessed August 30, 2024).

[B16] GBD 2019 Stroke Collaborators (2021). Global, regional, and national burden of stroke and its risk factors, 1990–2019: a systematic analysis for the Global Burden of Disease Study 2019. Lancet Neurol. 20, 795–820. doi: 10.1016/S1474-4422(21)00252-0 34487721 PMC8443449

[B17] Gubhaju B. Østbye T. Chan A. (2018). Living arrangements of community-dwelling older Singaporeans: predictors and consequences. Ageing Soc. 38, 1174–1198. doi: 10.1017/S0144686X16001495

[B18] Gyawali P. Chow W. Z. Hinwood M. Kluge M. English C. Ong L. K. . (2020). Opposing associations of stress and resilience with functional outcomes in stroke survivors in the chronic phase: a cross-sectional study. Front. Neurol. 11:230. doi: 10.3389/fneur.2020.00230 32390923 PMC7188983

[B19] Hadidi N. Treat-Jacobson D. J. Lindquist R. (2009). Poststroke depression and functional outcome: a critical review of literature. Heart Lung 38, 151–162. doi: 10.1016/j.hrtlng.2008.05.002 19254633

[B20] Hodkinson H. M. (2012). Evaluation of a mental test score for assessment of mental impairment in the elderly. Age Ageing 41(Suppl. 3), iii35–iii40. doi: 10.1093/ageing/afs148 23144286

[B21] Htun H. L. Wong L. H. Lian W. Koh J. Lee L. T. Lim J. P. . (2022). Functional improvement after inpatient rehabilitation in community hospitals following acute hospital care. Ann. Acad. Med. Singap. 51, 357–359. doi: 10.47102/annals-acadmedsg.2021507 35786756

[B22] Ifejika N. L. Awosika O. O. Black T. Duncan P. W. Harvey R. L. Katz D. I. . (2025). Improving access to stroke rehabilitation and recovery: a policy statement from the American Heart Association/American Stroke Association. Stroke 56, e218–e233. doi: 10.1161/STR.0000000000000493 40740119

[B23] Inouye M. Kishi K. Ikeda Y. Takada M. Katoh J. Iwahashi Y. . (2000). Prediction of functional outcome after stroke rehabilitation. Am. J. Phys. Med. Rehabil. 79, 513–518. doi: 10.1097/00002060-200011000-00007 11083301

[B24] Kalra L. (1994). Does age affect the benefits of stroke unit rehabilitation? Stroke 25, 346–351. doi: 10.1161/01.STR.25.2.346 8303743

[B25] Karatepe A. Günaydin R. Kaya T. Türkmen G. (2008). Comorbidity in patients after stroke: impact on functional outcome. J. Rehabil. Med. 40, 831–835. doi: 10.2340/16501977-0269 19242620

[B26] Kroenke K. Spitzer R. L. Williams J. B. W. (2003). The Patient Health Questionnaire-2: validity of a two-item depression screener. Med. Care 41, 1284–1292. doi: 10.1097/01.MLR.0000093487.78664.3C 14583691

[B27] Lehmann J. F. DeLateur B. J. Fowler R. S. Warren C. G. Arnhold R. Schertzer G. . (1975). Stroke rehabilitation: outcome and prediction. Arch. Phys. Med. Rehabil. 56, 383–389. 809023

[B28] MacKenzie D. M. Copp P. Shaw R. J. Goodwin G. M. (1996). Brief cognitive screening of the elderly: a comparison of the MMSE, AMT, and MSQ. Psychol. Med. 26, 427–430. doi: 10.1017/S0033291700034826 8685299

[B29] Mahoney F. I. Barthel D. W. (1965). Functional evaluation: the Barthel Index. Md. State Med. J. 14, 61–65. 14258950

[B30] Mayo N. E. Wood-Dauphinee S. Ahmed S. Carron G. Higgins J. McEwen S. . (1999). Disablement following stroke. Disabil. Rehabil. 21, 258–268. doi: 10.1080/096382899297684 10381238

[B31] Meyer M. J. Pereira S. McClure A. Teasell R. Thind A. Koval J. . (2015). A systematic review of studies reporting multivariable models to predict functional outcomes after post-stroke inpatient rehabilitation. Disabil. Rehabil. 37, 1316–1323. doi: 10.3109/09638288.2014.963706 25250807

[B32] Ministry of Health Singapore (2022). National One-Rehabilitation Framework. Available online at: https://www.hpp.moh.gov.sg/.../national-one-rehabilitation-framework.pdf (Accessed January 24, 2022).

[B33] Mutai H. Furukawa T. Araki K. Misawa K. Hanihara T. (2012). Factors associated with functional recovery and home discharge in stroke patients admitted to a convalescent rehabilitation ward. Geriatr. Gerontol. Int. 12, 215–222. doi: 10.1111/j.1447-0594.2011.00747.x 21929733

[B34] National Registry of Diseases Office (2022). Singapore Stroke Registry Annual Report 2019. Available online at: https://www.duke-nus.edu.sg/docs/librariesprovider3/research-policy-brief-docs/care-research-brief-6—caregivers-of-older-adults-in-singapore—an-overview-and-synthesis-of-empirical-studies-(online).pdf?sfvrsn=d3098e53_4 (Accessed January 21, 2022).

[B35] Ng Y. S. Tan K. H. X. Chen C. Senolos G. C. Chew E. Koh G. C. (2016). Predictors of acute, rehabilitation and total length of stay in acute stroke: a prospective cohort study. Ann. Acad. Med. Singap. 45, 394–403. doi: 10.47102/annals-acadmedsg.V45N9p394 27748786

[B36] O'Sullivan S. B. Schmitz T. J. (2022). Physical Rehabilitation, 5th Edn. Philadelphia, PA: F.A. Davis.

[B37] Otokita S. Uematsu H. Kunisawa S. Sasaki N. Fushimi Y. Imanaka Y. . (2021). Impact of rehabilitation start time on functional outcomes after stroke. J. Rehabil. Med. 53:jrm00145. doi: 10.2340/16501977-2775 33284355 PMC8772372

[B38] Peduzzi P. Concato J. Kemper E. Holford T. R. Feinstein A. R. (1996). Simulation study of the number of events per variable in logistic regression analysis. J. Clin. Epidemiol. 49, 1373–1379. doi: 10.1016/S0895-4356(96)00236-3 8970487

[B39] Pohjasvaara T. Vataja R. Leppävuori A. Kaste M. Erkinjuntti T. (2001). Suicidal ideas in stroke patients 3 and 15 months after stroke. Cerebrovasc. Dis. 12, 21–26. doi: 10.1159/000047676 11435675

[B40] Powers W. J. Rabinstein A. A. Ackerson T. Adeoye O. M. Bambakidis N. C. Becker K. . (2019). Guidelines for the early management of acute ischemic stroke: 2019 update. Stroke 50, e344–e360. doi: 10.1161/STR.0000000000000211 31662037

[B41] Riopel M. L. (2010). The Connor–Davidson Brief Resilience Scales. Positive Psychol. Available online at: https://positivepsychology.com/connor-davidson-brief-resilience-scale/ (Accessed January 30, 2022).

[B42] Serrano-Parra M. D. Garrido-Abejar M. Notario-Pacheco B. Bartolomé-Gutiérrez R. Solera-Martínez M. Martínez-Vizcaíno V. (2013). Validity of the CD-RISC-10 in non-institutionalized older adults. Enferm. Clin. 23, 14–21. doi: 10.1016/j.enfcli.2012.11.006 23352433

[B43] Shah S. Vanclay F. Cooper B. (1989). Improving the sensitivity of the Barthel Index for stroke rehabilitation. J. Clin. Epidemiol. 42, 703–709. doi: 10.1016/0895-4356(89)90065-6 2760661

[B44] Shin S. Lee Y. Chang W. H. Sohn M. K. Lee J. Kim D. Y. . (2022). Multifaceted assessment of functional outcomes in survivors of first-time stroke. JAMA Netw. Open 5:e2233094. doi: 10.1001/jamanetworkopen.2022.33094 36149652 PMC9508656

[B45] Simić-Panić D. (2018). The impact of comorbidity on rehabilitation outcome after ischemic stroke. Acta Clin. Croat. 57, 5–15. doi: 10.20471/acc.2018.57.01.01 30256006 PMC6400340

[B46] Singapore General Hospital (2022). Who We Are | Singapore General Hospital. Available online at: https://www.sgh.com.sg/about-sgh/who-we-are (Accessed January 28, 2022).

[B47] SingHealth Community Hospitals (2022). Corporate Profile | SingHealth. Available online at: https://www.singhealth.com.sg/sch/about-sch/corporate-profile (Accessed January 28, 2022).

[B48] Sloane K. L. Kasner S. E. Favilla C. G. Rothstein A. Witsch J. Hamilton R. H. . (2023). Always look on the bright side: associations of optimism with functional outcomes after stroke. J. Am. Heart Assoc. 12:e027959. doi: 10.1161/JAHA.122.027959 36870988 PMC10111448

[B49] Southwick S. M. Bonanno G. A. Masten A. S. Panter-Brick C. Yehuda R. (2014). Resilience definitions, theory, and challenges: interdisciplinary perspectives. Eur. J. Psychotraumatol. 5:25338. doi: 10.3402/ejpt.v5.25338 25317257 PMC4185134

[B50] Teasell R. W. Foley N. C. Bhogal S. K. Chakravertty R. Bluvol A. (2005). A rehabilitation program for patients recovering from severe Stroke. Can. J. Neurol. Sci. 32, 512–517. doi: 10.1017/s0317167100004534 16408584

[B51] UCHealth (2019). Why are Strokes on the Rise in Younger People? Available online at: https://www.uchealth.org/today/why-are-strokes-in-young-people-occurring-more-often/ (Accessed July 15, 2024).

[B52] Van De Weg F. B. Kuik D. J. Lankhorst G. J. (1999). Post-stroke depression and functional outcome: a cohort study investigating the influence of depression on functional recovery from stroke. Clin. Rehabil. 13, 268–272. doi: 10.1191/026921599672495022 10392654

[B53] Wang H. Camicia M. Terdiman J. Hung Y. Y. Sandel M. E. (2011). Time to inpatient rehabilitation hospital admission and functional outcomes of stroke patients. PM R. 3, 296–304. doi: 10.1016/j.pmrj.2010.12.018 21497314

[B54] World Health Organization (2022a). Stroke. Cairo: Eastern Mediterranean Regional Office. Available online at: http://www.emro.who.int/health-topics/stroke-cerebrovascular-accident/index.html (Accessed January 21, 2022).

[B55] World Health Organization (2022b). Social Determinants of Health. Available online at: https://www.who.int/health-topics/social-determinants-of-health (Accessed January 26, 2022).

[B56] Yoo J. Hong B. Jo L. Kim J. S. Park J. Shin B. . (2020). Effects of age on long-term functional recovery in patients with stroke. Medicina 56:451. doi: 10.3390/medicina56090451 32906615 PMC7558871

